# Neuropsychological outcome in children with optic pathway tumours when first-line treatment is chemotherapy

**DOI:** 10.1038/sj.bjc.6601410

**Published:** 2003-11-25

**Authors:** E Lacaze, V Kieffer, A Streri, C Lorenzi, E Gentaz, J-L Habrand, G Dellatolas, C Kalifa, J Grill

**Affiliations:** 1 Department of Pediatric and Adolescent Oncology, Gustave Roussy Institute, 39, rue Camille Desmoulins 94805, Villejuif, France; 2 Laboratory of Cognition and Development, CNRS-UMR 8605, René Descartes University, Henri Piéron Centre, Paris, France; 3 Centre Ressources, National Hospital of Saint-Maurice, Saint Maurice, France; 4 Laboratory of Experimental Psychology, CNRS-UMR 8581, René Descartes University, Henri Piéron Centre, Paris, Franace; 5 Department of Radiotherapy, Gustave Roussy Institute, Villejuif, France; 6 Laboratory of Epidemiology and Biostatistics, INSERM Unit U472, Villejuif, France

**Keywords:** child, neuropsychology, optic pathway glioma, radiotherapy, chemotherapy, visual acuity, blindness

## Abstract

Standard treatment of optic pathways gliomas consists of radiotherapy and surgery when feasible. Owing to the toxicity of irradiation, chemotherapy has emerged as an interesting therapeutic option, especially in young children. This study describes the neuropsychological profile of 27 children (aged between 1.5 and 15.7 years) with optic pathways gliomas treated with chemotherapy as first-line treatment. Eight of them also received radiotherapy as salvage treatment. Eight had neurofibromatosis type 1 (NF1). Intellectual outcome was preserved in children treated with chemotherapy only (mean=107±17) compared to children also receiving radiotherapy (mean IQ=88±24) or children having NF1 and treated with chemotherapy (mean IQ=80±13). Scores for abstract reasoning, mental arithmetic, chessboard/coding, perception, judgement of line orientation were lower in children irradiated than in those treated only by chemotherapy. Children with Nf1 showed subnormal IQ scores with marked impairment of short- and long-term memory. With respect to long-term neuropsychological outcome, our study shows that a chemotherapy-first strategy can preserve the intellectual outcome of these patients either by avoiding the need of radiotherapy or by delaying its use as much as possible.

Gliomas of the optic pathways (OPG) with or without contiguous involvement of the hypothalamus represent 5% of all childhood intracranial tumours (Heideman *et al*, 1993). The typical age of presentation in 70% of patients is within the first decade of life and in 90% within the first two decades. The mean age of presentation of patients with anterior visual pathway gliomas is 8.8 years (Dutton, 1994). Although these tumours are usually low-grade astrocytomas, their behaviour is highly variable, ranging from long-term stabilisation to progressive visual and neurological impairment culminating in death (Alvord and Lofton, 1988). Disease progression can be controlled by irradiation (Kovalic *et al*, 1990), but endocrinological and neurological late effects are severe (Cappelli *et al*, 1998). The principal sequelae of this tumour and its treatment consist of growth hormone deficiency and other endocrinological deficits (Brauner *et al*, 1990), cognitive deficits (Packer *et al*, 1983) and cerebrovascular complications (Grill *et al*, 1999). Moreover, when neurofibromatosis type 1 (NF1) is also present, one may encounter additional learning difficulties (Moore *et al*, 1994). Chemotherapy was introduced in the 1990 s to postpone or replace irradiation in the management of children with this type of tumour (Packer *et al*, 1997). This chemotherapeutic approach is expected to minimise the long-term side effects compared to irradiation.

The neuropsychological profile of children treated for an OPG has rarely been studied so far. Moreover, the benefit of a therapeutic strategy aimed at delaying or avoiding irradiation has not been demonstrated. The aim of this present study was to define patterns of neuropsychological deficits in children with optic pathway glioma treated with chemotherapy as first-line treatment as well as the risk factors for intellectual impairment (Laithier *et al*, 2003).

## MATERIALS AND METHODS

### Patients

All children treated first with BBSFOP chemotherapy at the Institut Gustave Roussy (IGR), Villejuif, France, for an optic pathway glioma were eligible for the study. Over a 1-year period, 27 children (11 boys and 16 girls) entered this cross-sectional study. The extent of the optic pathway tumour was classified according to Dodge classification (Dodge *et al*, 1958). Six patients had a Dodge II tumour, involving the optic chiasm and one or two optic nerves; 16 had a Dodge III tumour, involving the hypothalamus or adjacent structures. Eight had NF1 according to standard clinical criteria (National Institute of Health consensus conference, 1988). The patients' age ranged from 5 months to 8.5 years (median, 1.3 years) at tumour diagnosis, from 7 months to 9 years (median, 3 years) at first chemotherapy and from 1.5 to 15.7 years (median, 8.7 years) at the time of the neuropsychological assessment. Eight patients also received irradiation as part of the treatment of a relapse: their ages ranged from 4 to 9.11 years (median, 6.4 years) at radiotherapy. The interval between end of chemotherapy and the neuropsychological assessment ranged from 3 months to 9.4 years (median, 6.1 years) and from 1.1 to 5.8 years (median, 3.8 years) for children treated with radiotherapy.

### Treatment

Figure 1

**Figure 1:**
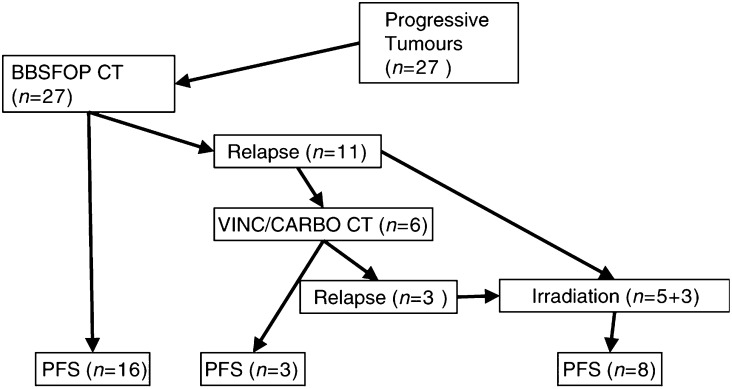
Distribution of patients according to treatments. PFS=progression-free survivors; VINC=vincristine; CARBO=carboplatin; BBSFOP CT=BBSFOP chemotherapy, that is, multiagent regimen of six drugs over 16 mo.

describes the therapeutic sequences used in the patients who entered this study. The BBSFOP protocol consists of seven cycles of three different courses combining either carboplatin or natulan (course A), etoposide and cisplatin (course B) or vincristine and cyclophosphamide (course C). The whole chemotherapy is given over a period of one and a half years. No irradiation was planned at the end of chemotherapy. In case of relapse, second-line chemotherapy could be administered, usually the vincristine–carboplatin chemotherapy as previously described (Packer *et al*, 1997). Salvage treatment with irradiation had to be postponed as much as possible, preferably after the age of 4 years. Local irradiation was given with two beams lateral to the tumour volume with a security margin as described previously (Cappelli *et al*, 1998) until 1996, and thereafter conformal techniques with multiple beams were used (Table 1

**Table 1:** Radiotherapy techniques in eight children receiving irradiation as salvage treatment after failure of chemotherapy

**Patients**	**Age at diagnosis**	**Age at irradiation**	**Beams**	**Technique**	**Dose (Gy)**	**Last FSIQ (interval since radiotherapy)**
1	8.5	10	4 (2 antero-post, 2 lateral)	Photons 18 MeV	52	124 (5.5 years)
2	4	7	2 lateral	Photons 4.5/18 MeV	50	82 (5 years)
3	2.5	6.5	2 lateral	Co60/Photons	50	81 (1 year)
4	3.5	6.5	4 (2 antero-post, 2 lateral)	Photons 18 MeV	50	76 (2 years)
5	1.5	4.5	4 (2 antero-post, 2 lateral)	Photons 18 MeV	50	56 (2 years)
6	1.5	6.5	4 non co-planar	Photons 18 MeV	50	98^a^(3 years)
7	1	5.5	4 non co-planar	Photons 18 MeV	50	63^b^(2 years)
8	0.5	4	8 iso-centric	Photons 18 MeV	50	81 (2 years)

Age is given in years. MeV=mega-electron volt (high-energy photons); Gy=grey.

a Results of the last evaluation (i.e. 3 years after the end of radiotherapy). For the analysis of the results of the whole group, the previous results at 2 years were used.

b Estimation of the full-scale IQ since the patient could not perform all the visual task due to severely impaired vision.

).

### Neuropsychological assessment

The neuropsychological assessment protocol included a uniform request for evaluation of intellectual ability. Owing to the range of patients' ages at the time of assessment, intellectual functioning was assessed using either the Brunet-Lezine (1997) Revised (for children less than 3 years old, *n*=6), or the age-appropriate Wechsler scale (WPPSI-R for children less than 7 years old, *n*=5 children; WISC-III for children over 7 years old, *n*=16 children) (Wechsler, 1989, 1991). Laterality was determined using the Dellatolas protocol (De Agostini and Dellatollas, 1988). Short-term memory was explored by the digit span (De Agostini *et al*. 1999). Long-term memory was explored by the Rey auditory verbal learning test (Rey, 1941). Oral language was evaluated by the verbal fluency and vocabulary subtests from the WISC III. All test scores were standardised to a mean of 10 and an s.d. of 2 using available age norms, except for the IQ in which the scores were already standardised to a mean of 100 and an s.d. of 15. The visual agnosia was explored by the recognition of 80 pictures from the Snodgrass test (Snodgrass and Vandervart, 1980) and the shape-recognition test of the KABC (Kaufman and Kaufman 1983) (scores like Wechsler scale). For the Snodgrass test, scores were given as a percentage of recognised pictures: at 5 years, 100% of the pictures should be recognised. The visual-spatial skill was assessed with the Judgment of Line Orientation tests (Lindgren and Benton, 1980) and scores were given in s.d. using available age norms. The visual tests were used only in children with a preserved vision, that is, at least four out of 10 for the best eye.

To examine perceptual skills in sensory modalities other than vision, we created a test of tactile recognition of 10 known and 10 unknown objects. This test explores the cross-modal transfer between touch and vision (Streri, 1993). Scores were the number of correct answers. We also explored auditory skills with a task of intensity discrimination and a task of modulation detection threshold. A personal computer controlled the auditory measures. All stimuli were delivered binaurally via an earphone at a level of 75 dB SPL. Thresholds were obtained using an adaptative two-interval, two-alternative forced-choice adaptative procedure to evaluate the intensity or modulation necessary for 70.7% correct detection (Hall and Grose, 1990). The latter two tests were standardised on children at school.

The tests were timed, and a 3-h period was necessary for the entire evaluation. Tests were always performed in the same order. Some children were unable to perform any visual test, and they just performed verbal and auditory tests. Information regarding school placement, both before disease onset and at the time of neuropsychological assessment, was also collected from interviews with the parents.

### Factors evaluated for impact on intellectual outcome

Socio-economic status (SES) was estimated from the parents' professions and categorised into two groups: low/average and high (Mueller and Parcel, 1981). The visual acuity was determined in all patients. Other risk factors studied were age at diagnosis, presence of NF1, treatment with radiotherapy and tumour size according to Dodge staging (Dodge *et al*, 1958).

### Statistical analysis

Statistical analysis was performed using Statistica software (StatSoft, Guyancourt, France, 1996). Group performances were compared using analysis of variance (ANOVA). Significant ANOVA tests were followed up with a *t-*test in which the *α* levels were set at 0.05. This method requires a significant *F* value for the overall ANOVA and is considered to be the most powerful of the common *post hoc* multiple comparison procedures. Percentages were compared with the *χ*^2^ test. Pearson product–moment correlations were computed in determining the relationship between medical variables and neuropsychological performance.

## RESULTS

### Study of very young children

Six children 17 months to 3.4 years old were assessed using the Brunet-Lezine Revised scale. None of them had been irradiated before evaluation. The median quotient was 83 (range 70–101) for full-scale development quotient (DQ), 100 (range 74–109) for postural development, 77 (range 52–87) for oculomotor development, 82.5 (range 66–139) for language development and 82.5 (range 79–109) for socialisation. Ocular-motor DQ was the most impaired in each child, while the other domains showed high interindividual differences. For language skills, the children were on average 6 months behind. The three children with NF1 had the worst full-scale DQ. One of these patients had been blind since the age of 3 months and showed global psychomotor developmental retardation. At the time of the neuropsychological evaluation, none of the children was attending school.

### Study of older patients

A total of 21 children 4.3–15. 7 years old (median 8.7 years) passed the WISC-III scale. The results of the neuropsychological assessment in these 21 older patients were as follows: 10 had a full-scale intelligence quotient (FSIQ) greater than 85, that is, in the normal range; eight had an FSIQ between 70 and 85; and three had an FSIQ below 70. The mean FSIQ, the verbal IQ (VIQ) and the performance IQ (PIQ) were 92 (median 83), 97 (median 98) and 88 (median 86), respectively.

The VIQ scores of most children were usually above their PIQ scores, with a statistically significant difference of 9 points (*P*=0.02). The mean results of the WISC III performance subtests were around 7 (i.e. below the normal range), with a marked drop for picture arrangement and object assembly.

Treatment with radiotherapy and the presence of NF1 were the two risk factors for neuropsychological impairment. There was no statistically significant correlation for the main neuropsychological scores and the SES, age at diagnosis, extent of disease judged with the Dodge stage and visual acuity. Verbal IQ, however, was slightly lower in children with larger tumours (Dodge III *vs* Dodge II, *P*=0.05). Thus, the impact of radiotherapy and NF1 was analysed by comparing the following three subgroups: treatment with chemotherapy only and absence of NF1 (Group 1, *n*=8), chemotherapy followed by radiotherapy and absence of NF1 (Group 2, *n*=8) and chemotherapy in children with NF1 (Group 3, *n*=5). Full-scale IQ scores were more often in the normal range in non-NF1 children treated with chemotherapy only; 87.5% had an IQ over 85 in group 1, 25% in group 2 and 20% in group 3, respectively (*P*=0.016, *χ*^2^ test). General intellectual functioning was normal and higher among non-NF1 children treated with chemotherapy alone than among those also receiving irradiation or who had NF1 (median, 106 *vs* 81 *vs* 77, respectively) (see Table 2

**Table 2:** Results of Wechsler scales in the three groups

**Scores nmean (s.d.)**	**Group 1 Chemoth.**	**Group 2 Radioth.**	**Group 3 NF1**	**Statistically significant differences**
*IQ scales*				
Full scale	107 (16.9)	87.6 (23.6)	80.4 (13.3)	G1 *vs* G3 (*P*=0.03)
Verbal	108.6 (11.9)	91 (24.8)	88.2 (19.4)	NS
Performance	102 (18.9)	83.4 (19.2)	76 (11.7)	G1 *vs* G3 (*P*=0.02)
Indices				
Comprehension	106.3 (10.6)	92.4 (23.3)	77.5 (0.7)	NS
Perception	109.5 (16.2)	86 (17.1)	81 (5.6)	G1 *vs* G2 (*P*=0.04)
Speed	102.5 (16.1)	84.2 (16.5)	82.5 (12)	G1 *vs* G2 (*P*=0.04)
*Subtests of verbal scale*				
Information	10.9 (2.9)	10.1 (5)	8.2 (3.5)	NS
Similarities	12.6 (2.3)	7.4 (5)	8.8 (2.3)	G1 *vs* G2 (*P*=0.009)
Arithmetic	11.5 (3.5)	7.7 (4.7)	7 (4.5)	NS
Vocabulary	12.6 (4.6)	8 (3.8)	8.2 (3.7)	G1 *vs* G2 (*P*=0.008), G1 *vs* G3 (*P*=0.02)
Comprehension	10.2 (4.6)	8.9 (2.9)	8.2 (3.8)	NS
Digit span	10.6 (3.1)	9 (1.7)	4.3 (0.6)	G1 *vs* G3 (*P*=0.001), G2 *vs* G3 (*P*=0.008)
*Subtests of performance scale*				
Picture completion	12.3 (4.5)	9.3 (4.9)	7.6 (3)	NS
Block designs	12.6 (4)	8.7 (3.1)	7 (2)	G1 *vs* G2 (*P*=0.04), G1 *vs* G3 (*P*=0.01)
Object assembly	9.3 (2.2)	6.7 (2.8)	4.6 (2.9)	G1 *vs* G3 (*P*=0.009)
Chessboard/coding	10.6 (2.1)	6.3 (4.4)	10 (2.6)	G1 *vs* G2 (*P*=0.05)
Mazes	8.2 (3.9)	5.5 (4)	6.4 (4.1)	NS
Picture arrangement	8.5 (4.8)	6.3 (3)	4.7 (0.6)	NS
Symbols	9.5 (3.8)	7 (2.7)	5.4 (2.3)	G1 *vs* G3 (*P*=0.04)

; Figure 2

**Figure 2:**
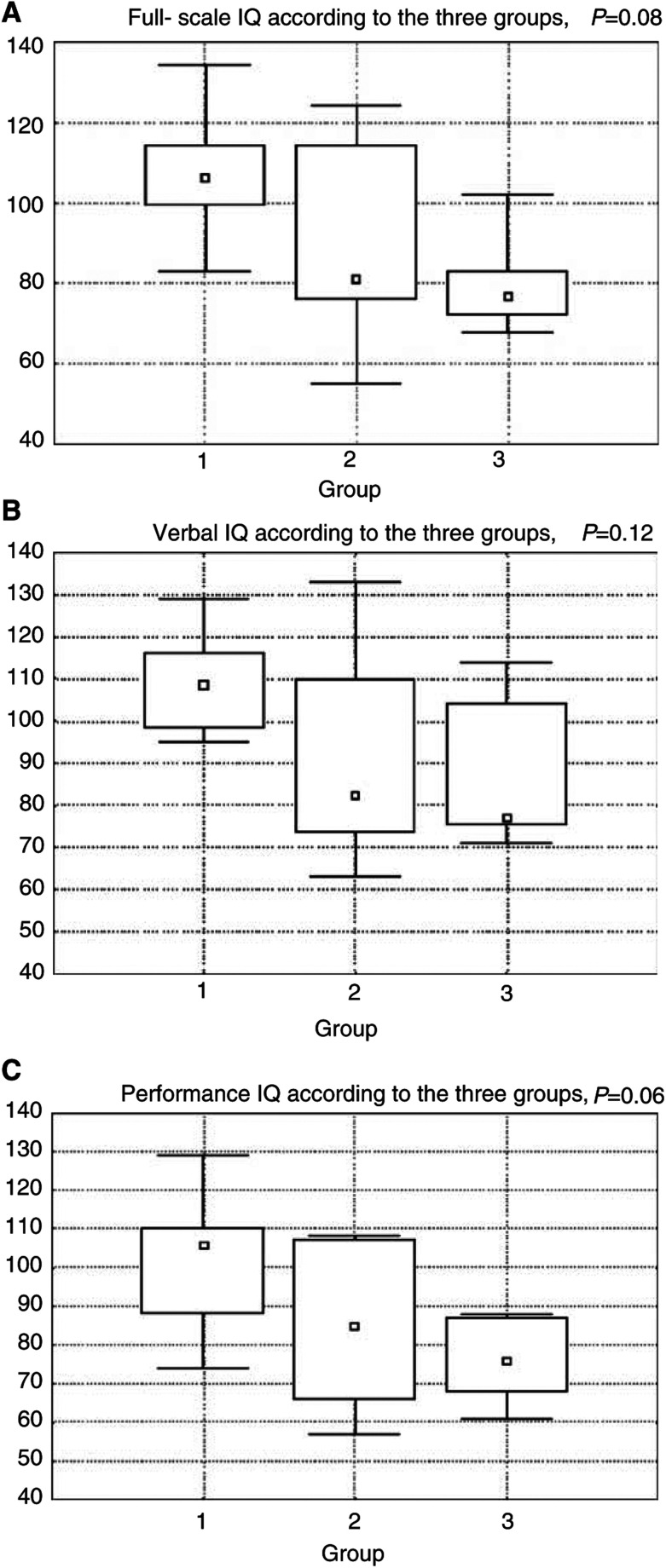
IQ scores of the three groups: (**A**) full scale IQ; (**B**) verbal IQ; (**C**) performance IQ. Scores represent the median value of the distribution. Boxes represent 25–75% of the distribution. Bars represent the range, that is, minimal and maximal values. Group 1=patients without NF-1 treated with chemotherapy only, Group 2=patients without NF1 treated with chemotherapy followed by radiotherapy, Group 3=patients with NF1 treated with chemotherapy only.

).

For the verbal scale, children who received radiotherapy had significantly more difficulties in abstract reasoning and vocabulary than children treated only by chemotherapy (7.3 *vs* 12.6, *P*=0.009; 8 *vs* 12.6, *P*=0.008, respectively). They also had more difficulties in arithmetic, but the difference did not reach statistical significance (*P*=0.09). Digit span performance was significantly lower in children with NF1 than in children treated only with chemotherapy (4.3 *vs* 10.5, *P*=0.001) or radiotherapy (4.3 *vs* 9, *P*=0.008). Moreover, children with NF1 had difficulties in mental arithmetic because of their poor working memory. Like the children treated with radiotherapy, they tended to have more difficulties in abstract reasoning and significantly for vocabulary than children treated only with chemotherapy (8.8 *vs* 12.6, *P*=0.07; 8.2 *vs* 12.6, *P*=0.02, respectively).

For the performance scale, three children (one in group 1, two in group 2) could not perform the test because of their poor visual acuity. The children who received radiotherapy tended to have more difficulties with chessboard/coding, object assembly and had significantly more difficulties with block designs than children treated only with chemotherapy (6.2 *vs* 10.6, *P*=0.05; 6.7 *vs* 9.3, *P*=0.09; 6.7 *vs* 9.3, *P*=0.04; respectively). Children treated with radiotherapy had impaired visual-constructive abilities and slowness. Symbols were also low (7 *vs* 9.5). The children with NF1 showed markedly impaired ability in every subtest of the performance scale except for chessboard/coding (mean score=10), unlike the group of children treated with radiotherapy. Block designs and object assembly performance were significantly lower in children with NF1 than in children treated only by chemotherapy (7 *vs* 12.6, *P*=0.01; 4.6 *vs* 9.3, *P*=0.009). Three children passed the neuropsychological evaluation before and after radiotherapy. Two remained with stable IQ during the first 2 years after irradiation and one had a clear deterioration (−13 points 4 years after irradiation).

### Results of the other tests performed in older children

In every other area assessed, the mean score of the children in group 1 was higher than in groups 2 and 3 (Table 3

**Table 3:** Complementary tests in the three groups

**Scores Mean (SD)**	**Group 1 Chemoth.**	**Group 2 Radioth.**	**Group 3 NF1**	**Statistically significant differences**
List of words				
Immediate recall	0.45 (1.3)	−0.33 (1.2)	−1.04 (0.8)	NS
Delayed recall	0.5 (0.8)	7.4 (13.4)	20.3 (11.6)	G1vsG3 (p=0.01)
Recognition	0.07 (1.3)	0.20 (0.6)	0.16 (0.4)	NS
Digit span				
Digit Span Direct	0.24 (0.9)	−0.24 (0.9)	−0.30 (1.2)	NS
Digit Span Indirect	−0.07 (1.4)	−0.76 (1.6)	−1.40 (0.6)	NS
Snodgrass test ^*^	90.9 (6.3)	92.2 (8.7)	83.4 (18.2)	NS
Benton line	0.13 (1)	−1.35 (2.1)	−1.35 (0.9)	NS
Verbal fluency	1.71 (1.3)	−0.67 (1.5)	0.07 (1.8)	G1vsG2 (p=0.05)
Shape recognition test	7.6 (3.4)	6 (4.3)	7.2 (2.9)	NS
Tactile Recognition				
Test A	8.9 (1.5)	9.2 (0.8)	6.3 (2)	G1vsG3 and G2vsG3(p=0.01)
Test B	5.8 (1.8)	8.8 (1.5)	5.5 (0.7)	G1vsG2 (p=0.04)

*P*-values correspond to a Student's is *t*-test performed as *post hoc* analysis when ANOVA analysis indicated a statistically significant difference between the three groups. NS=nonsignificant; s.d.=standard deviation. Results of the Snodgrass pictures recognition test is given as the percentage of good responses that should be 100% at 5 years. This test was only rated for children over 5 years.

).

Short-term memory, explored by the digit span method, differed significantly in the three groups (*P*=0.004). Immediate recall was normal in groups 1 and 2, with respective mean scores of 0.45s.d. and −0.33s.d., and a low/normal range in group 3 (−1.04s.d.). Delayed recall was more deficient than immediate recall in groups 2 and 3, with respective mean percentages of omission of 7.39 and 20.3%. Delayed recall was significantly lower in group 3 than in group 1 (0.48 *vs* 20.3%, *P*=0.01). Recognition was normal for the three groups (mean 0.14s.d.).

Performance in the Judgment of Line Orientation test was poor and similar in groups 2 and 3 (−1.35 s.d.) relative to group 1 (0.13s.d.), but the difference was not statistically significant. Recognition of 80 pictures in the Snodgrass test correlated with visual acuity of the best eye (*r*=0.50, *p*=0.05). Considering that 100% of the pictures are recognised by children of 5 years and above, performance was impaired for the three groups (91, 93, 83% correct answers, for groups 1, 2 and 3, respectively), with a marked drop for the animal category (mean score of 86%). Verbal fluency was lower in group 2 than in group 1 (1.71 *vs* –0.67s.d., *P*=0.05). Shape recognition was impaired in the three groups (7.6, 6, 7.2 for groups 1, 2 and 3 respectively), with no statistically significant difference.

The tactile recognition test was performed by 15 children. Tactile recognition of known objects was significantly different (*P*=0.03) in the three groups. Patients in group 3 exhibited difficulty in recognition of known and unknown objects.

The auditory tasks were performed by 10 children. Six children showed normal results on the two tasks. Two children did not succeed in performing the tasks and two children, one in group 2 and one in group 3, showed an impaired detection of modulation associated with a normal discrimination threshold. This profile is in agreement with a possible temporal central deficit. These two patients had verbal deficit and a poor comprehension of language.

School attendance at the time of the neuropsychological evaluation was normal in 11 children, while 10 children went to specialised schools for the blind and visually disabled. School attendance was not statistically different in the three groups. Five children had psychomotor therapy for the re-education of space orientation. Seven had speech therapy (one in group 1, three in group 2 and three in group 3).

## DISCUSSION

The purpose of this study was to describe the neuropsychological deficits in children with optic pathway glioma whose first-line adjuvant treatment was chemotherapy, and to evaluate the effects of salvage treatment with radiotherapy. Although these encouraging results need to be confirmed in future multicentre studies, one can consider that the goal of improving the intellectual outcome by a chemotherapy-first study compared to irradiation (Cappelli *et al*, 1998; Fouladi *et al*, 2003) is achievable.

The non-NF1 children treated only with chemotherapy showed a normal mean full-scale IQ (score=107). The mean VIQ was above the normal range, while the mean PIQ was slightly impaired. In most studies of radiotherapy, a significant proportion of children had severely impaired cognitive skills (Packer *et al*, 1983; Silber *et al*, 1992; Capelli *et al*, 1998). In a recent study of 31 children with OPT, half of them treated with radiotherapy, the mean full-scale IQ was subnormal (score = 86) at diagnosis and remained stable during follow-up (Fouladi *et al*, 2003).

As expected, visual-spatial skills were the most affected by the disease and its treatment. The scores for picture recognition (Snodgrass test) were correlated with the extent of the visual deficit.

Even after treatment without irradiation, NF1 children with OPT have a dismal neuropsychological prognosis. They have difficulties with abstract reasoning, vocabulary and digit span in the verbal scale and block designs, object assembly and symbols in the performance scale. They also show a deficit in judgement of line orientations, in short- and long-term memory as well as in visual recognition. Studies of specific learning disability in children with NF1 show intellectual impairment in as many as 40% of the children in some series (Riccardi, 1992; North *et al*, 1994). Deficits are apparent in academic achievement (spelling, arithmetic and reading), language, visual-spatial and concentration abilities as in our study. Eliason (Eliason, 1986) indicated that most of the children with NF1 in his sample did not differ from the general population in terms of verbal IQ, but had a significantly lower performance IQ. His sample was found to have an extremely high rate of visual-perceptual disability without evidence of OPT. To understand the relative contribution of neurofibromatosis and brain tumour to the cognitive profile of children with neurofibromatosis, Moore *et al*, (1994) studied the neuropsychological deficits of children with neurofibromatosis, brain tumour or both. The mean scores of the neurofibromatosis–brain tumour group were generally the lowest of the three groups; those of the brain tumour group were highest, and performance of the neurofibromatosis group was generally between the other two groups. The results indicate an increased incidence of cognitive impairment and learning disability in children with NF1 and suggest that the diagnosis of NF1 itself, especially in the context of a brain tumour, is associated with a high incidence of meaningful neuropsychological deficits.

The full-scale IQ in irradiated children was lower than in nonirradiated children. Such children had difficulties with abstract reasoning and vocabulary in the verbal scale and block designs, object assembly and slowness on chessboard/coding on the performance scale. Their results in delayed recall of a list of words and in judgement of line orientation and verbal fluency are lower than in children treated only by chemotherapy. However, one must consider that children receiving irradiation in our cohort had more aggressive form of OPT with at least one relapse after chemotherapy that prompted us to use radiotherapy. Consequently, they do not represent a real control group for children treated with chemotherapy only. Nevertheless, several studies on children surviving medial edge intracranial tumours who underwent brain irradiation showed a deterioration in a number of specific cognitive functions (Kun and Mulhern, 1983; Packer *et al*, 1983; Bendersky *et al*, 1988; Duffner *et al*, 1988; Morrow *et al*, 1989; Garcia-Perez *et al*, 1994): the most affected items were memory followed by attention, sequential processing and visual-spatial organisational skills. In the study of Fouladi, preschool children (i.e. before the age of 5) had a worse intellectual outcome than older children: mean IQ 79 *vs* 96, respectively, *P*=0.003. Thus, our results in patients treated with chemotherapy first compare favourably with those obtained in children of similar age treated with radiotherapy (mean IQ of our whole cohort=92 compared with 79 in the study of Fouladi). One can hope that more sophisticated irradiation techniques such as conformal radiotherapy, intensity-modulated radiotherapy or proton beam irradiation will cause less cognitive damage.

The results in all 21 patients show a dissociation between a relatively preserved verbal IQ and an impaired performance IQ because the tumours were on the optic pathway, which would influence the visual recognition and discrimination required to perform some of the items on the performance subscale of the Wechsler Intelligence for Children-Revised. The patients exhibited poor object recognition, sometimes due to impaired visual acuity. Thus, the diagnosis of visual agnosia cannot be clearly demonstrated.

For the very young children, the Brunet-Lezine scale revealed oculomotor deficit that is worsened by impaired visual acuity. Psychomotor development, language and socialisation were in the normal range in the absence of NF1.

Tactile and auditory tests were created to observe the skills in other modalities and to find another way to evaluate children with visual deficits. The cross-modal transfer task had showed a good preservation of tactile recognition except for NF1 children, which can be used for rehabilitation. Studies (Rose and Feldman, 1995; Rose *et al*, 1998) have shown that this task is correlated with IQ and we can see that children with NF1 who had difficulties in this task had also the lowest IQ.

The auditory task was also successfully completed by all children except for two who showed a central auditory deficit (Hall and Grose, 1994; Lorenzi, 1999), which may explain their verbal deficit and a bad comprehension of language (Cacace and MacFarland, 1998). In fact, speech intelligibility depends heavily on the accurate perception of auditory temporal envelope cues, which is the slower amplitude modulations present in the speech waveform (Hall and Grose, 1994; Hescot *et al*, 2000; Lorenzi *et al*, 2000).

It is thus important to obtain information with these tests in every patient for the design of specific and individualised rehabilitation programmes.

## CONCLUSION

The neuropsychological outcome of children with optic pathway gliomas treated by chemotherapy is preserved. Children whose disease needed to be treated with irradiation had a worse neuropsychological outcome, but the effect of irradiation cannot easily be discriminated in this study from the effect of a more severe disease course. We consider that a thorough neuropsychological evaluation is required, together with evaluation of visual acuity and fields in the assessment of children treated in prospective trials. In addition to their accuracy in discriminating treatment and comorbidity effects, these evaluations may help to define individualised rehabilitation programmes.
